# The Future and the Nation's Choice

**Published:** 1918-11-23

**Authors:** 


### THE FUTURE AND THE NATION'S CHOICE.

Though history marks its days of celebration and pauses to survey and sum up the past, time itself knows no halting-place, and thus it is that even the crowded and poignant memories of the last four years do not exempt us from the necessity of facing and calculating the future. That our calculations cannot possess an absolute quality is no doubt true, for, as we are somewhat cynically told, it is the unexpected that happens. Still, we have to use the best judgment we can command, and, though this may not rule the future, it will certainly influence it. Here lies a responsibility which may not be escaped, and with it is associated a degree of power and of opportunity sufficient to stir to enthusiasm and to what is still better—namely, effort and determination. Leaving aside elements which defy estimate, it is not too much to say that the future of the nation will depend upon a sound judgment of values, and this consideration must weigh on all those whose duty it is to endeavour, whether by spoken or by written word, to contribute to the development and organisation of public opinion and decision. Hardly may we expect to escape entirely either the breezes of sentimentality or the gusts of passion, and for these some allowances must be made. But safety is to be secured mainly by the cultivation and exercise of a sound judgment, and we would fain hope that to this faculty our national leaders will address their appeal. The journalist speaks to a smaller audience, but he is not less a sharer in the main responsibility. It is right and seemly that the achievements of the past should be recalled both in justice to those who have fought and laboured and died to accomplish them and for purposes of encouragement and high hope. The chronicle and preservation of the record are secure beyond doubt. But the current page has rather the present and the future for its text, and it is here that arises a public responsibility and obligation. None may avoid the pressure of the bond, and the measure of opportunity is the measure also of duty.

In spite of uncertainties, there is surely ground for confidence in the light of recent experiences. With what sure judgment did our people approve the entry of the nation on a war for the defence of public law and the pledged word, and how completely have events justified the decision! In spite of losses and griefs that may not be described, is there anyone who now regrets the clear vision of that day of fate? Not less significant is the history which shows a resolution determined to persevere to the appointed end. The judgment has certainly not on all sides been a careful calculation of chances and probabilities. In measure, indeed, it has been a non-conscious process. But it has been sound and wise and enduring, and, however compassed, it merits a liberal recognition. Perhaps the problems of the immediate future are not so simple or so clean-cut as the issue of peace or war. But the same courage and goodwill which solved the one will be addressed to the other, and, though there may at times be dark days, the face of the nation, we are sure, will be turned towards the light.

